# Lightning Stroke

**Published:** 1859-08

**Authors:** 


### LIGHTNING STROKE.

It is said that exposure to the rain or being drenched with buckets of water, seem to have some agency in restoring persons to life who have been prostrated by lightning.

It is better to take some precautions against the lightning, which will be the more easily remembered, and the better applied if some explanations are given as to the nature of lightning.

There is a stillness in the atmosphere when all parts of it are of equal temperature, whether cold or hot, for the air is then in equilibrium. But if one part be hot, and the other be cold, as in two adjoining rooms, the moment the door between is opened there is a commotion, and the cold air rushes into the warmer room.

If two vessels of water adjoin and are connected by a hollow tube under the surface, both bodies of water are still, if each vessel is filled to an equal height. But if one vessel has a greater depth of water than the other, there is a commotion until an equilibrium is secured.

When the atmosphere about us is uniformly filled or saturated with electricity, there is quiet, safety, equilibrium. But if a layer either side has more or less electricity than the one about us, there is a passing of the electricity from one to the other, until each body of air is alike filled or equally saturated. But with this passing there is noise, as the passing of air makes the noise of wind, and the passage of water causes roaring, so the noise made by the passage of electricity is called thunder; the force of it is the lightning, as the force of wind or moving water carries us away, according to its rapidity; but lightning, like a cannon ball, moves so swiftly that the body which it strikes has not time to have motion imparted to it, and it is shivered or perforated; the comparison how-does not hold good at all points.

But the electricity of the fuller section or body of air gets to the other which has less, with greater or less facility according to what is between them, or connects them. If a pointed piece of metal, gold, silver or iron connects these bodies of different fullness of electricity, the communication or stream is conducted so constantly and steadily, that there is no noise or commotion, there is no obstruction. But if wood is used, it does not conduct the electricity quick enough; hence wood is not as good a conductor as iron. Hence where there is more electricity above us than on the earth, it comes down quietly and unnoticed if there are a great many iron communications or conductors, such as lightning rods; but if trees only, extend from one to the other, or tall chimneys, there is noise and destruction. Hence it is best to keep away from chimneys and trees, or tall objects in thunder storms in warm weather; therefore if in the house, keep as near the centre of the room as possible.

But the course or direction of the lightning is always from the fuller air to that which is less full, as water runs from the fuller vessel towards the other. Hence if the air in the clouds has most electricity, the "stroke" comes from above; if however the air on the surface is fuller of electricity then the stroke is upwards; this is the reason in many cases, why men and animals are killed by lightning in the open fields, or on prairies.

But these unequally filled bodies of air may be parallel with each other, and if a house is between them, it will be a conductor, and a person sitting at an open window will be killed: if the window had been down, he might have been saved, for glass repels lightning; that is, it can keep it from passing; hence if a man stands on the ground and takes hold of an electrical wire, the electricity will pass freely through his body into the earth; but if he stands on a glass block, the electricity does not go through, but collects in the man himself; he gets full of it, and "fire flies" out of him every time you touch him.

Lightning or electricity has a love, so to speak, for metals, has an affinity for them, or seeks for them, hence the less of iron, or steel, or other metals, you have about your person during a thunder storm in summer, the safer you are.

